# Amikacin pharmacokinetic/pharmacodynamic in intensive care unit: a prospective database

**DOI:** 10.1186/s13613-020-00685-5

**Published:** 2020-06-08

**Authors:** Elsa Logre, Maya Enser, Sébastien Tanaka, Marie Dubert, Aurore Claudinon, Nathalie Grall, Hervé Mentec, Philippe Montravers, Olivier Pajot

**Affiliations:** 1 CH Argenteuil, réanimation polyvalente, 69 rue du Lieutenant-Colonel Prudhon, Argenteuil, France; 2 CHU Bichat, réanimation chirurgicale, Paris, France; 3 INSERM UMR1188 Diabète - Athérothrombose - Thérapies Réunion Océan Indien (DéTROI), Saint-Denis de la Réunion, Université de la Réunion, Réunion, France; 4 CHU Bichat, maladies infectieuses et tropicales, Paris, France; 5 CH Argenteuil, microbiologie, Argenteuil, France; 6 CHU Bichat, microbiologie, Paris, France

**Keywords:** Pharmacokinetic, Pharmacodynamic, Amikacin, Intensive care unit

## Abstract

**Background:**

Aminoglycosides have a concentration-dependent therapeutic effect when peak serum concentration (*C*_max_) reaches eight to tenfold the minimal inhibitory concentration (MIC). With an amikacin MIC of 8 mg/L, the *C*_max_ should be 64–80 mg/L. This objective is based on clinical breakpoints and not on measured MIC. This study aimed to assess the proportion of patients achieving the pharmacokinetic/pharmacodynamic (PK/PD) target *C*_max_/MIC ≥ 8 using the measured MIC in critically ill patients treated for documented Gram-negative bacilli (GNB) infections.

**Methods:**

Retrospective analysis from February 2016 to December 2017 of a prospective database conducted in 2 intensive care units (ICU). All patients with documented severe GNB infections treated with amikacin (single daily dose of 25 mg/kg of total body weight (TBW)) with both MIC and *C*_max_ measurements at first day of treatment (D1) were included. Results are expressed in n (%) or median [min–max].

**Results:**

93 patients with 98 GNB-documented infections were included. The median *C*_max_ was 55.2 mg/L [12.2–165.7] and the median MIC was 2 mg/L [0.19–16]. *C*_max_/MIC ratio ≥ 8 was achieved in 87 patients (88.8%) while a *C*_max_ ≥ 64 mg/L was achieved in only 38 patients (38.7%). Overall probability of PK/PD target attainment was 93%. No correlation was found between *C*_max_/MIC ratio and clinical outcome at D8 and D28.

**Conclusion:**

According to PK/PD parameters observed in our study, single daily dose of amikacin 25 mg/kg of TBW appears to be sufficient in most critically ill patients treated for severe GNB infections.

## Key points

- A single daily dose of amikacin (25 mg/kg of TBW) appears to be sufficient in most critically ill patients treated for severe GNB infections.
- An exclusive focus on *C*_max_ without MIC measurement is probably not suitable for a reliable pharmacodynamic assessment of amikacin therapy.

## Background

Management of severe infections in intensive care unit (ICU) represent a major challenge for clinicians, and prompt initiation of effective antibiotic therapy is essential to improve patient’s survival [1, 2]. In patients with septic shock, guidelines recommend combination antimicrobial therapy with aminoglycosides, particularly when Gram-negative bacilli (GNBs) are suspected or documented [2].

Optimal anti-GNB activity of amikacin is achieved when peak serum concentration (*C*_max_) reach eight to tenfold the minimal inhibitory concentration (MIC) [3]. With an amikacin MIC as high as 8 mg/L (beyond which the bacteria have intermediate susceptibility based on clinical breakpoints defined by EUCAST), the target *C*_max_ should therefore be 64–80 mg/L as recommended by French guidelines on aminoglycosides use [4, 5].

Previously published data with single daily dose of 15 to 25 mg/kg of amikacin pointed out that this target of *C*_max_ ≥ 64 mg/L was not reached for most patients [6–8]. Thus, in order to optimize the *C*_max/_MIC ratio, it is suggested to increase amikacin loading dose up to 30 mg/kg/day of total body weight (TBW) for severe patients [5].

However, in most clinical studies and in daily practice, the pharmacological efficacy of amikacin is only assessed by *C*_max_ measurements (objective of *C*_max_ ≥ 64 mg/L based on clinical breakpoints) and not by measured MICs.

To our knowledge, few studies have reported the antibacterial effect of amikacin based on *C*_max_/MIC ratio using measured MIC for each patient profile.

We therefore undertook a retrospective analysis of our prospective database to assess pharmacodynamic target attainment (*C*_max_/MIC ≥ 8)—considering amikacin measured MICs—in critically ill patients empirically treated for documented severe GNB infections.

## Methods

### Study design, settings and patients

We performed a retrospective analysis from February 2016 to December 2017 of a prospective observational database conducted in 2 ICUs.

The ANSM (French National Agency for Medicines and Health Products Safety) registration number of the study is 2017-A01083-50. French Data Protection Agency declaration of the database was done. The local hospital ethics Committee was consulted and did not indicate the need for a formal approval, according to French law [9].

ICU patients who met the following criteria were included: (i) empirical combination treatment including amikacin for sepsis or septic shock (as defined by the 3rd Surviving Sepsis Campaign) [2], with a diagnosis made according to CDC infections classifications [10]; (ii) amikacin administered according to the standardized protocol of both ICUs (described below) and following microbiological sample for pathogen identification and (iv) documented GNB infection with amikacin MIC measurement available.

Patients with the following criteria were excluded: (i) death within 48 h after amikacin administration; (ii) no GNB documented in microbiological sample; (iii) incorrect amikacin regimen; (iv) resistance to amikacin on antibiogram; (v) no MIC measurement available.

### Protocol

Patients were receiving empiric antibiotic therapy with amikacin in combination with one (or more) other antimicrobial agents, whose choice was left to the discretion of clinicians.

Amikacin was administered according to the standardized protocol of both ICUs (in place since 2015): recommended single daily dose of 25 mg/kg of TBW (weight of the day, using a weighing bed), diluted in 50 mL NaCl 0.9% and continuously infused (electric syringe) over 30 min.

The duration of treatment with amikacin was left to the discretion of clinicians.

According to the standardized protocol, peak amikacin concentration was measured 30 min after the end of infusion (*C*_max_) and trough serum concentration 24 h after the end of infusion and before the next injection of amikacin, if necessary (*C*_min_).

Reinjection was not recommended if *C*_min_ was beyond 2.5 mg/L, according to national guidelines [5].

When GNB was identified, clinicians could ask the microbiology laboratory of each hospital to perform amikacin MIC measurement for patients who received amikacin. Measurement was performed using a diffusion technique in an agar medium (Etest strips) for each strain according to the manufacturer’s instructions (bioMérieux laboratory, Marcy l’étoile, France). The toxicology laboratory performed amikacin serum concentrations measurements as a routine procedure available 24 h a day, 7 days a week, using a fluorescence polarization immunoassay (FPIA) [11].

### Objectives of the study and endpoints

The main objective of the study was to determine the proportion of patients achieving a *C*_max_/MIC ratio ≥ 8, using both MIC and *C*_max_ measurements at first day of treatment.

In case of a polymicrobial infection, the highest amikacin MIC among identified GNBs was considered.

The secondary objectives were to describe amikacin pharmacological parameters; to determine the overall probability of pharmacodynamic target attainment in patients treated with a 25 mg/kg daily dose of amikacin, taking into account the distribution of *C*_max_ and MIC; to identify covariables of amikacin *C*_max_ in critically ill patients. We also evaluated the impact of amikacin pharmacokinetic/pharmacodynamic (PK/PD) parameters on clinical outcome. Poor clinical outcome was defined as a composite criteria: SOFA score > 3 or death at D8.

### Data collection

D1 was defined as the first day of amikacin administration (calendar day).

For all patients enrolled, demographic data, Simplified Acute Physiology score (SAPS 2) [12], SOFA score (Sequential Organ Failure Assessment) [13], reason for ICU admission, type of infection, and clinical and biological parameters were retrospectively collected on D1 and D8.

Daily fluid intake on D1, vasopressor support, duration of mechanical ventilation, use of renal replacement therapy (RRT), of Optiflow^®^ or ECMO (extracorporeal membrane oxygenation) were noted, if applicable.

Patient vital status was assessed on D8 and D28.

### Statistical analysis

Variables were expressed as numbers (percentages) and medians [minimal–maximal].

Patients or infections were compared according to different pharmacokinetic and pharmacodynamic parameters (*C*_max_, *C*_max_/MIC ratio) on D1, and for the clinical outcome evaluated on D8 and D28. A non-parametric test was used to compare the continuous variables (Mann–Whitney test), and an appropriate test was used to compare the categorical variables (Chi-square test or Fisher’s exact test).

Variables associated with *C*_max_ ≥ 64 mg/L in univariate analysis were included in a multivariate analysis using stepwise logistic regression.

A p value less than 0.05 was considered statistically significant.

The overall probability of pharmacodynamic target C_max_/MIC ratio ≥ 8 was calculated by adding the individual probabilities for each observed MIC value, taking into account the frequency of observation of each MIC and the observed *C*_max_ distribution.

The statistical analyses were performed using R software (Team RC) and JMP 14.1 (SAS Institute Inc, Cary, NC, USA http://www.R-projectorg/).

## Results

### Patients characteristics

**Fig. 1:**
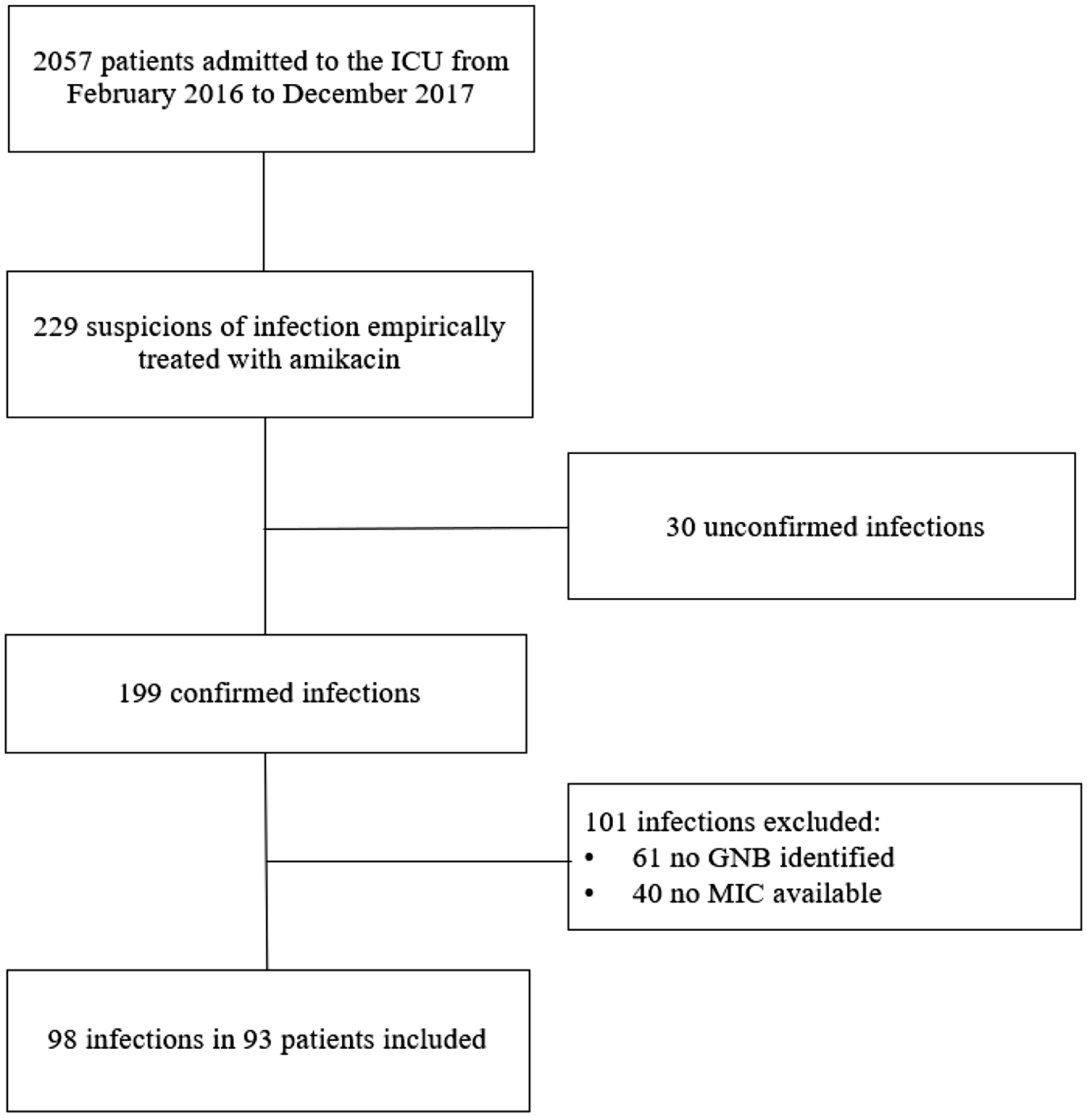
Flowchart of the study

From February 2016 to December 2017, 93 patients with 98 GNB infections were eligible (Fig. 1). Patients characteristics at admission are reported in Table 1.

**Table 1:** Patients and infections characteristics on admission and on D1

Patients characteristics on admission	Values (*n *= *93*)
Age (years)	62 [24–90]
Female	37 (40)
BMI (kg/m^2^)	25 [15–45]
Chronic renal failure	17 (18.2)
SAPS 2 admission	54 [15–124]
Types of admission	
Medical	52 (56)
Scheduled surgery	13 (14)
Urgent surgery	28 (30)
Reasons for admission	
Septic shock	36 (39)
Acute respiratory failure	30 (30.6)
Heart failure	8 (8.1)
Pulmonary transplantation	8 (8.1)
Other	16 (16.2)

Clinical characteristics on D1	Values (*n *= *98*)
Delay admission–D1 (day)	4.5 [1–71]
Weight (kg)	77 [45–130]
Temperature (°C)	37.8 [35–41]
Leukocytes (G/L)	14.3 [0.1–78.7]
Creatinine (µmol/L)	77 [23–609]
SOFA score	7 [0–17]
Vasopressors	58 (59.1)
Mechanical ventilation	62 (63.3)
Infections characteristics	
Types of infection	
Pulmonary	52 (53)
Intra-abdominal	24 (24.5)
Urinary	15 (15.3)
Cutaneous	3 (3.1)
Other	4 (4)
Bacteriemia	14 (14.3)
Polymicrobial infections	46 (46.7)
Gram-negative bacilli documented (*n* = 132)	
*E. coli*	41 (31.1)
*P. aeruginosa*	39 (29.5)
*E. cloacae*	10 (7.6)
*K. pneumoniae*	11 (8.3)
*Morganella spp*	8 (6.1)
*P. mirabilis*	5 (3.8)
*C. koseri*	5 (3.8)
Others	13 (9.8)
Bacteria other than GNB identified	35 (35.7)

Data presented in *n* (%) or median [min–max]

The clinical characteristics of patients and infections on D1 are reported in Table 1.

A β-lactam (4th generation cephalosporin or piperacillin–tazobactam in 49% and carbapenem in 32.6% of infections) was used in combination with amikacin in 97 infections (99%). Empiric antibiotic therapy was active against the pathogen(s) in 87 infections (89%) among which amikacin was the only active antibiotic in only 5 (5.1%) infections.

Amikacin median MIC for all GNBs identified in our study was 2 mg/L [0.19–16] and the median MIC of *Pseudomonas aeruginosa* was 3 mg/L [1–8]. Two infections involved bacteria with amikacin MIC of 12 mg/L and 16 mg/L, respectively (intermediate susceptibility according to EUCAST).

MICs distribution of amikacin is available in (Additional file 1: Fig. S1).

### Primary outcome

The *C*_max_/MIC ratio, calculated for each infectious episodes on D1, was above or equal to 8 in 87 infectious episodes (88.8%) (Table 2). Excluding the two infections involving GNB with intermediate susceptibility according to EUCAST, which usually discourage amikacin prescription, a *C*_max_/CMI ratio ≥ 8 was achieved in 91% of treated infections.

**Table 2:** Pharmacokinetic/pharmacodynamic parameters observed

PK/PD parameters of amikacin	
Total administered dose (mg)	1900 [1000–3250]
Dose administered by body weight on D1 (mg/kg)	25 [15.6–31.8]
Time [end of infusion−*C*_max_ measurement] (min)	30 [10–55]
*C*_max_ D1 (mg/L)	55.2 [12.2–165.7]
*C*_max_ D1 ≥ 64 mg/L	38 (38.8)
*C*_max_ D1 ≥ 80 mg/L	18 (18.4)
*C*_max_/MIC on D1	23.1 [1–169]
*C*_max_/MIC ≥ 8 on D1	87 (88.8)
*C*_max_/MIC ≥ 10 on D1	86 (87.8)
*C*_min_ D2 ≥ 2.5 mg/L^a^	49 (59.8)

Data presented in *n* (%) or median [min–max]

^a^Among the 82 (84%) *C*_min_ available on D2

### Secondary outcomes

#### Pharmacological parameters

The PK/PD parameters of amikacin are reported in Table 2. The median *C*_max_ on D1 was 55.2 mg/L [12.2–165.7]. In 38.8% of infectious episodes, amikacin *C*_max_ was ≥ 64 mg/L on D1.

Pharmacodynamic description was also performed including the following criteria: *C*_max_ ≥ 80 mg/L and *C*_max_/MIC ratio ≥ 10 (Table 2).

**Fig. 2:**
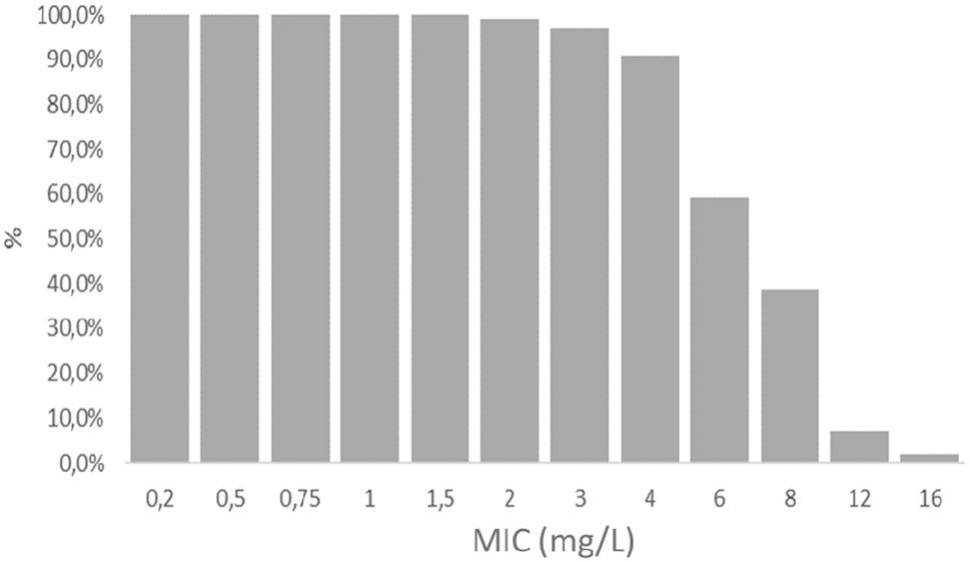
Probability of achieving target *C*_max_/MIC ratio ≥ 8 according to the MIC

The overall probability of target attainment *C*_max_/MIC ≥ 8, with single daily dose of 25 mg/kg amikacin under the study conditions, according to *C*_max_ and MICs distributions, was 93% (Fig. 2). This probability was 89% for a target of *C*_max_/MIC ≥ 10.

GNBs infections treated with a *C*_max_/CMI ratio < 8 versus ≥ 8 were associated with both higher amikacin MICs (8 [4–16] versus 2 mg/L [0.19–8], *p* < 0.0001) and lower *C*_max_ on D1 (34.2 [12.2–77.8] versus 57.6 mg/L [22.3–165.7], *p* = 0.001) (Additional file 1: Table S1).

#### *C*_max_ covariables

Factors associated with a *C*_max_ ≥ 64 mg/L in univariate analysis are reported in Table 3. In multivariate analysis, after adjustment with the SOFA score on D1 and the administered dose, serum creatinine on D1 was the only factor independently associated with a *C*_max_ ≥ 64 mg/L (OR = 1.01 [1.00–1.01], *p* = 0.004).

**Table 3:** Comparison of patients and treated infections, according to the 1st *C*_max_ observed (on D1)

Patients data^a^	*C*_max_ on D1		*p*
	< 64 mg/L	≥ 64 mg/L	
	*(n *= *56)*	*(n *= *37)*	
Female	20 (35.7)	17 (45.9)	0.32
Age (years)	60 [24–90]	66 [46–88]	0.038
BMI admission	24.5 [15–42.4]	25.4 [18.8–45.1]	0.17
SAPS 2 admission	51.5 [15–105]	54 [29–124]	0.38
Chronic renal failure	7 (12.5)	10 (27)	0.10

Data on D1	(*n* = 60)	(*n* = 38)	
Admission time—D1 (d)	5 [1–71]	4 [1–35]	0.25
Weight (kg)	75.5 [45–130]	79.5 [48–120]	0.15
Weight gain/admission (kg)	0 [− 15–25]	0 [− 12–23]	0.26
SOFA	6.5 [0–17]	8 [0–15]	0.17
Mechanical ventilation	41 (68.3)	21 (55.2)	0.19
Vasopressor support	32 (53.3)	26 (68.4)	0.14
Renal replacement therapy	2 (3.3)	3 (7.9)	0.37
Urine 24 h volume (mL)	1100 [0–5300]	1225 [0–4900]	0.52
Serum creatinine (µmol/L)	58.5 [23–453]	132 [31–609]	0.0003
24-h fluid intake (mL)	3200 [1000–12,642]	2868 [1000–11,000]	0.90
Dose administered per kg (mg)	25 [15.6–31.8]	25 [20.3–31.3]	0.11
Total dose administered (mg)	1800 [1000–3250]	2000 [1250–3000]	0.034

Data expressed in n (%) or median [min–max]

^a^Comparison according to the *C*_max_ measured during the first episode of infection treated with amikacin

#### Clinical outcome

The median length of stay in intensive care unit was 16 days [2–123]. Overall mortality at D28 was 24.7% and ICU mortality was 22.6%.

There was no significant differences in mortality and SOFA score on D8 according to the *C*_max_/MIC ratio. Similarly, the clinical outcome of patients on D28 was not different (Table 4).

**Table 4:** Comparison of patients clinical outcome and treated infections, according to *C*_max_/MIC ratio observed < or ≥ 8 on D1

Overall population		*C*_*max*_*/*MIC on D1		*p*
		<* 8*	≥* 8*	
Patients^a^	*(n *= 93)	*(n *= 10)	*(n *= 83)	
Length of stay in intensive care unit (d)	16 [2–123]	16 [2–123]	16 [2–96]	0.80
Mortality D28	23 (24.7)	2 (20)	21 (25.3)	1
Infectious episodes	*(n *= 98)	*(n *= 11)	*(n *= 87)	
Mortality D8	11 (11.2)	2 (18.2)	9 (10.3)	0.60
SOFA D8	3 [0–12]	2 [0–6]	3 [0–12]	0.75

Data expressed in *n* (%) or median [min–max]

^a^Comparison according to the *C*_max_/MIC ratio observed during the first episode of infection treated with amikacin

PK/PD parameters were not different whether the clinical outcome was favorable or poor on D8 (Additional file 1: Table S2), and similarly between patients alive or dead on D28 (Additional file 1: Table S3).

## Discussion

In this prospective database performed in critically ill patients with documented GNB infections and receiving a 25 mg/kg single daily dose of amikacin, the overall probability of target attainment *C*_max_/MIC ratio ≥ 8 was 93% according to our *C*_max_ and MIC distributions.

These data are not in accordance with previous studies that showed a risk of treatment failure, only based on amikacin *C*_max_ measurement (64 to 80 mg/L) or *C*_max_/MIC ratio calculated with clinical breakpoints (8 to 10-fold a 8 mg/L MIC) [7, 8]. Most studies concluded that a 25 mg/kg loading dose of amikacin is insufficient in critically ill patients to achieve these pharmacodynamic targets and therefore endanger treatment efficacy.

However, in our study, although only 38.8% patients only achieved an amikacin *C*_max_ ≥ 64 mg/L, more than 90% had a *C*_max_/MIC ratio over the pharmacodynamic target (≥ 8) when susceptible strains were involved.

Amikacin *C*_max_ measurements ranged from 12.2 to 165.7 mg/L with a median of 55.2 mg/L. This significant dispersion found in several studies conducted in ICUs is explained by the inter-individual variability of pharmacokinetic parameters in these patients [7, 8, 14–16].

Nevertheless, in our study, the median amikacin *C*_max_ was much lower than in the literature (summarized in Additional file 1: Table S4). Indeed, three studies performed in critically ill patients and receiving a 25 mg/kg (TBW) loading dose reported a median amikacin *C*_max_ around 70 mg/L [7, 8, 14]: De Montmollin et al. showed that 58% of patients enrolled reached an amikacin *C*_max_ ≥ 64 mg/L [7] and Taccone et al. reported 70% of patients achieving this target [8].

Patients characteristics in our study and those reported above seem similar especially in terms of severity (estimated by the SOFA score), but there are significant differences in renal function (renal replacement therapy resort and serum creatinine were lower in our patients). These differences are all the more important as serum creatinine is a covariable of the pharmacokinetic of aminosides, which seems decisive for *C*_max_. Indeed, to our knowledge, two studies identified renal function as a major co-variable of amikacin *C*_max_ in ICU patients and this may explain the lower amikacin *C*_max_ found in our study [14, 17]. We confirm these results because, in multivariate analysis, after adjustment with the SOFA score on D1 and the administered dose, serum creatinine on D1 was the only factor independently associated with a *C*_max_ ≥ 64 mg/L (OR = 1.01 [1.00–1.01], p = 0.004).

In any event, it is even more striking to reach about 90% of PK/PD target attainment with these lower amikacin *C*_max_, which seems related to low amikacin MICs.

Amikacin median MICs of all GNBs identified in our study was 2 mg/L [0.19–16] and the median MIC of *Pseudomonas aeruginosa* was 3 mg/L [1–8]. These results were comparable to amikacin MICs distribution for GNB published by EUCAST and in the literature [18, 19].

Unsurprisingly, in the group of infectious episodes that did not reach the *C*_max_/CMI ratio target, the median MIC of amikacin was higher (8 mg/L [4–16]). But excluding intermediate susceptibility GNBs, a *C*_max_/CMI ratio ≥ 8 was achieved in 91% of treated infections.

Few other studies reported inconsistencies between calculated *C*_max_ or *C*_max_/MIC (using clinical breakpoints) and measured *C*_max_/MIC (using measured MICs). A study based on measured MICs (for some patients enrolled), reported a *C*_max_/MIC ratio ≥ 10 in 93% of cases, with a median loading dose of 29.6 mg/kg, while target amikacin *C*_max_ was only achieved in 77% of cases [20]. Similarly, Pajot et al. reported that *C*_max_/MIC ratio ≥ 10 was achieved in 87% patients with documented ventilator-associated pneumonia treated with only 20 mg/kg amikacin [18]. Finally, De Winter et al. in an emergency department, in which a cohort of patients with septic shock/severe sepsis received 25 mg/kg amikacin, showed that *C*_max_/MIC ratio ≥ 8 was achieved in 76% of cases when using critical MICs (EUCAST), and in 95% of cases when using measured MICs [21].

These findings suggest that an exclusive focus on target *C*_max_ without MIC measurement is probably not suitable to evaluate the pharmacodynamic efficacy of amikacin therapy in clinical studies. Thus, added value of daily practice amikacin pharmacodynamic assessment is also uncertain, as the probability of pharmacodynamic failure to treat susceptible GNBs with a 25 mg/kg amikacin dose is less than 10% when evaluated by the measured *C*_max_/MIC ratio in several different studies.

Nevertheless, when the MIC is above 4 mg/L, the risk of pharmacological failure is higher (around 40%) and clinicians should keep in mind the key role of the MIC, particularly when *Pseudomonas aeruginosa* is involved. Thus, dose adjustment could be relevant, based on local epidemiology (MICs distribution and/or *P. aeruginosa* infections incidence).

Increasing AMK loading dose could, however, lead to withhold the subsequent doses, if needed. Indeed, we reported a *C*_min_ ≥ 2.5 mg/L—when measured on D2—in 49/82 (60%) of treated infections and Roger et al. found similar results with a 30 mg/kg loading dose (*C*_min_ ≥ 2.5 mg/L in 49% of their patients) [20].

No correlation was found between *C*_max_/MIC ratio and clinical outcome on D8 or D28 in our study. Moore et al. and Kashuba et al. demonstrated, 30 years ago, that the clinical benefit of aminoglycosides for the treatment of GNB infections was optimal if *C*_max_ reach 8 to tenfold the MIC [3, 22]. But recent studies in ICU patients have failed to demonstrate a potential impact of aminoglycoside pharmacodynamics on clinical outcome [7, 8, 20, 23]. This negative result should be put into perspective with the various variables that may possibly explain mortality in intensive care. The combination with another antibiotic, most often a β-lactam, could also explain the lack of demonstration.

Even so, the median *C*_max/_MIC ratio in our study greatly exceeded the threshold proposed by Moore et al. [3] (23.1 versus 8), but the proportion of pharmacodynamic failure according to the following criteria (*C*_max_/MIC < 8) was low, around 10%, which represents a small cohort for statistical comparisons.

Our study has several limitations. First, it is a retrospective study, based on a prospective database in 2 participating ICUs and in daily practice, BGN-documented infections are not systematically assessed with measured amikacin MIC. Factors that led to measure the MIC could introduce a selection bias, leading to measure the MIC only in the most difficult clinical situations. Nevertheless, the patients evaluated in our study are similar to those evaluated by De Montmollin et al. and Taccone et al. (in term of SAPS II score and mortality), allowing indirect comparison of our data with the literature [7, 8]. Second, we did not assess the incidence of renal toxicity following amikacin administration. However, 64% of infections were treated with single daily dose of amikacin, minimizing the risk of nephrotoxicity. In addition, data from the literature do not report an increase in renal toxicity following the initial dose [24]. Gàlvez et al. also showed that a dose of 30 mg/kg/day amikacin was not associated with a higher incidence of nephrotoxicity than 15 or 25 mg/kg/day regimen [6]. Finally, we studied the pharmacodynamic profile of amikacin during the first 24 h of administration. This is consistent with usual durations of aminoglycoside treatments (48 to 72 h) and with data reported by Kashuba et al. [22] that highlighted the impact of the first aminoglycoside dose on patient’s clinical outcome.

## Conclusion

In critically ill patients treated with amikacin for susceptible Gram-negative bacilli infections, a 25 mg/kg (TBW) single daily dose actually achieved pharmacodynamic target in more than 90% of treated infections. Thus, in light of these results, the current trend of amikacin increase dose in the treatment of severe infections does not appear justified when local GNB ecology and amikacin MICs distribution are similar to ours. The relevance of systematic *C*_max_ measurement is also questionable or may require MIC measurement as well to ensure a reliable pharmacodynamic target assessment.

## Supplementary information

**Additional file 1: Table S1.** Comparison of patients and treated infections based on the *C*_max_/CMI ratio. Data presented in n (%) or median [min–max]. **Table S2.** Comparison of patients and treated infections according to clinical outcome on D8 evaluated by SOFA score. Data presented in n (%) or median [min–max]. **Table S3.** Comparison of patients and infections treated according to mortality on D28. Data presented in n (%) or median [min–max]. **Table S4.** PK/PD parameters observed in intensive care unit (recent studies). **Figure S1.** MICs distribution of all bacteria identified and considered responsible for infections

## Data Availability

The datasets used and analyzed during the current study are available from the corresponding author on reasonable request.

## Abbreviations

BMI: Body mass index
*C*_max_: Peak serum concentration
*C*_min_: Minimum concentration
EUCAST: European Committee on Antimicrobial Susceptibility Testing
GNB: Gram-negative bacilli
ICU: Intensive care unit
MIC: Minimal inhibitory concentration
PD: Pharmacodynamic
PK: Pharmacokinetic
SAPS 2: Simplified Acute Physiology Score
SOFA: Sequential Organ Failure Assessment
TBW: Total body weight
