# Supplementary material for: Amikacin pharmacokinetic/pharmacodynamic in intensive care unit: a prospective database
Source: Ann Intensive Care. 2020 Jun 8;10:75. doi: 10.1186/s13613-020-00685-5 (PMC7276966; doi:10.1186/s13613-020-00685-5)

**Additional file**

Table S1. Comparison of patients and treated infections based on the C_max_/CMI ratio. Data presented in n (%) or median [min-max].

|  | *C_max_ /CMI on D1* | |  |
| --- | --- | --- | --- |
|  | *< 8* | *≥ 8* | *p* |
| *Patients data** | *(n=10)* | *(n=83)* |  |
| Age (ans) | 62 [41–85] | 62 [24–90] | 0.89 |
| Female (%) | 3 (30) | 34 (41) | 0.73 |
| BMI (kg/m^2^) | 25.2 [15–30.8] | 25 [15–45.1] | 0.72 |
| SAPS 2 | 61 [31–89] | 52 [15–124] | 0.32 |
| *Data on D1* | *(n=11)* | *(n=87)* |  |
| Admission time– D1 (d) | 10 [1–71] | 4 [1–44] | 0.04 |
| Weight (kg) | 78 [45–96] | 76 [45–130] | 0.78 |
| Leucocytes (G/L) | 17.2 [10–33] | 14 [0.1–79] | 0.09 |
| *P. aeruginosa* infection | 4 (36) | 32 (37) | 1 |
| Total dose administered (mg) | 1850 [1000–2400] | 1900 [1000–3250] | 0.79 |
| Dose administered per kg (mg/kg) | 24.8 [22.2–26.4] | 25 [15.6–31.8] | 0.22 |
| MIC (mg/L) | 8 [4–16] | 2 [0.19–8] | <0.0001 |
| C_max_ (mg/L) | 34.2 [12.2-77.8] | 57.6 [22.3–165.7] | 0.001 |
| Acute renal failure | 4 (36.4) | 37 (42.5) | 0.75 |
| Serum creatinine (µmol/L) | 72 [23–436] | 80 [27–609] | 0.70 |
| Urine 24h volume (mL) | 1000 [0–3200] | 1200 [0-5300] | 0.73 |
| 24-hour liquide supply (mL) | 3200 [1500–10397] | 3000 [1000–12642] | 0.83 |
| SOFA | 4 [1–17] | 7 [0–15] | 0.42 |
| Vaopressor support | 5 (45.4) | 53 (60.9) | 0.35 |
| Mechanical ventilation | 6 (54.5) | 56 (64.4) | 0.52 |
| Renal replacement therapy | 1 (9.1) | 4 (4.6) | 0.45 |
|  |  |  |  |
| * : comparison according to C_max_/CMI measured during the first episode of infection treated with amikacin | | | |

Table S2. Comparison of patients and treated infections according to clinical outcome on D8 evaluated by SOFA score. Data presented in n (%) or median [min-max].

|  | *Outcome on D8* | |  | |
| --- | --- | --- | --- | --- |
|  | *SOFA score ≤ 3* | *SOFA > 3 or death* | | *p* |
| *Patients** | *(n=47)* | *(n=46)* | |  |
|  |  |  | |  |
| Age (years) | 63 [24–90] | 60 [30–85] | | 0.22 |
| Female (%) | 22 (47) | 15 (32.5) | | 0.16 |
| BMI (kg/m^2^) | 25.4 [18–45.1] | 24.7 [15–42.4] | | 0.96 |
| SAPS 2 | 51 [15–95] | 55 [27–124] | | 0.06 |
| *Data on D1* | *(n=49)* | *(n=49)* | |  |
| Admission time - D1 | 2 [1–35] | 6 [1–71] | | 0.01 |
| *P. aeruginosa* infection | 15 (30.6) | 21 (43) | | 0.20 |
| *Infected site* |  |  | | 0.10 |
| Pulmonary | 22 (45) | 30 (61) | |  |
| Extra-pulmonary | 27 (55) | 19 (39) | |  |
| Single dose of amikacin D1-D4 | 34 (69) | 29 (59) | | 0.29 |
| SOFA | 5 [0–15] | 9 [1–17] | | <0.0001 |
| Use of vasopressors | 24 (49) | 34 (69) | | 0.04 |
| Mechanical ventilation | 24 (49) | 38 (77.5) | | 0.003 |
| Renal replacement therapy | 1 (2) | 4 (8) | | 0.36 |
| Serum creatinine (µmol/L) | 73 [27–541] | 80 [23–609] | | 0.38 |
| C_max_ (mg/L) | 59.7 [12.2–165.7] | 55.2 [22.3–126.1] | | 0.16 |
| C_max_/MIC | 26.1 [1–169.9] | 22.9 [3.6–75.2] | | 0.16 |
|  |  |  |  | |
| *: comparison according to SOFA calculated at D8 during the first episode of infection treated with amikacin | | | | |

Table S3. Comparison of patients and infections treated according to mortality on D28. Data presented in n (%) or median [min-max].

|  | *Clinical outcome on D28* | |  |
| --- | --- | --- | --- |
|  | *Death (n=23)* | *Alive (n=70)* | *p* |
| *Patients* |  |  |  |
| Age (years) | 70 [51–85] | 59.5 [24–90] | <0.0001 |
| Female (%) | 7 (30.4) | 30 (43) | 0.29 |
| BMI (kg/m^2^) | 25 [15–39.3] | 25.4 [15–45.1] | 0.96 |
| SAPS 2 | 55 [27–124] | 51.5 [15–95] | 0.09 |
| *Data on D1** |  |  |  |
| Admission time - D1 | 5 [1–19] | 3 [1–71] | 0.57 |
| *P. aeruginosa* infection | 8 (35) | 25 (36) | 0.93 |
| *Infected site* |  |  | 0.58 |
| Pulmonary | 13 (56.5) | 35 (50) |  |
| Extra-pulmonary | 10 (43.5) | 35 (50) |  |
| Single dose of amikacin D1-D4 | 7 (30) | 32 (46) | 0.19 |
| SOFA | 10 [2–17] | 6.5 [0–15] | 0.0008 |
| Use of vasopressors | 19 (83) | 36 (51) | 0.08 |
| Invasive mechanical ventilation | 17 (74) | 41 (58.5) | 0.01 |
| Renal replacement therapy | 1 (4.3) | 3 (4.3) | 1 |
| Serum creatinine (µmol/L) | 109 [42–390] | 69.5 [27–609] | 0.012 |
| C_max_ (mg/L) | 59.7 [25.9–84.6] | 55.2 [12.2–165.7] | 0.68 |
| C_max_/MIC | 30.1 [4.7–75.2] | 23.1 [1–168.9] | 0.5 |
| *Data on D8** |  |  |  |
| SOFA | 5.5 [1–12] | 2 [0–12] | 0.01 |
| * : data observed during the first episode of infection treated with amikacin | | | |

Table S4. PK/PD parameters observed in intensive care unit (recent studies).

| References | Allou *et al*. [16] | Taccone *et*  *al*. [8] | | De Montmollin *et al.* [7] | Boidin *et al.*[14] | Roger *et al.* [20] |
| --- | --- | --- | --- | --- | --- | --- |
| Total dose (mg) | 2100 [1800-2400] | | 1750 [1125- 3000] | 1900 [1600-2250] | 1986 (±512) | ND |
| Dose (mg/kg) | 30 [29.2-30.6] | | 25 | 25 [24.6-25.5] | 25.7 (±5.1) | 29.6 (±3.3) |
| C_max_ (mg/L) | 75 [66.1-86.1] | | 72.7 [61.7-90.2] | 69 [54.9-84.4] | 73.0 (±23) | 75.8 (±24.5) |
| C_max_ ≥ 64mg/L | 81.80% | | ND | 58% | 63.10% | >77% |
| C_max_/MIC | NA | | 9.6 ± 3.5* | NA | NA | 94% >10** |
| Results in median [IQR], mean (± SD), or %  *: MIC EUCAST (8mg/L); **: 16 MIC available in 47 evaluated episodes | | | | | | |

Figure S1: MICs distribution of all bacteria identified and considered responsible for infections.


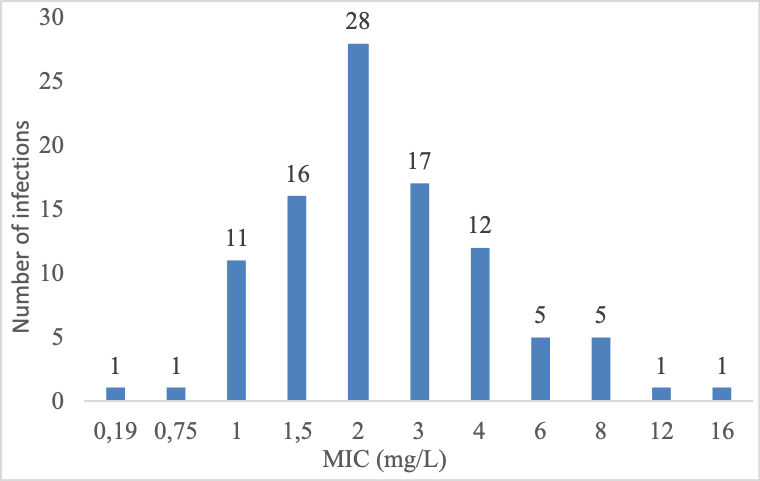

Supplement: Supplementary file 1 — Additional file 1: Table S1. Comparison of patients and treated infections based on the Cmax/CMI ratio. Data presented in n (%) or median [min–max]. Table S2. Comparison of patients and treated infections according to clinical outcome on D8 evaluated by SOFA score. Data presented in n (%) or median [min–max]. Table S3. Comparison of patients and infections treated according to mortality on D28. Data presented in n (%) or median [min–max]. Table S4. PK/PD parameters observed in intensive care unit (recent studies). Figure S1. MICs distribution of all bacteria identified and considered responsible for infections [file 13613_2020_685_MOESM1_ESM.doc]
